# Descriptive and psychometric properties of the Persian version of the Weiss functional impairment rating scale: parent report form in Iranian children

**DOI:** 10.1186/s12955-018-1053-1

**Published:** 2018-12-07

**Authors:** Behnaz Kiani, Habib Hadianfard, Margaret D. Weiss

**Affiliations:** 1 Department of Clinical Psychology, School of Educational Sciences and Psychology, Shiraz University, Eram Square, Shiraz, Iran; 2 Cambridge Health Alliance, Harvard University, Cambridge, MA USA

**Keywords:** Children, Functional impairment, Weiss functional impairment rating scale-parent report form, Psychometric properties

## Abstract

**Background:**

This study evaluates the descriptive and psychometric properties of the Persian version of the Weiss Functional Impairment Rating Scale-Parent Report Form (WFIRS-P) in a normal sample of Iranian children.

**Method:**

Parents of 282 students (grades 1–6) completed the WFIRS-P. Means and standard deviations were computed for the total scale, each domain, and each item of the WFIRS-P. Internal consistency, interdomain correlations, and test-retest reliability were used to assess the reliability of the scale.

**Results:**

Among the WFIRS-P domains, life skills had the highest rated impairment (*M* = 0.50, *SD* = 0.37) and risky activities had the lowest. Internal consistency (α = .88) and test-retest reliability (*r* = 0.77) were strong for the WFIRS-P total scale. The correlation between the WFIRS-P domains and the total scale ranged from 0.52 to 0.81.

**Conclusions:**

Results suggest that the Persian version of the WFIRS-P is a useful and psychometrically reliable measure for assessing functional skills in children.

## Background

Functional impairment is defined as significant reduction in social, school, family and other domains. Functional impairment can lead to certain limitations in an individual’s daily activities as the consequences of a disorder [1, 2]. The Diagnostic and Statistical Manual of Mental Disorders, Fifth Edition (DSM-5) requires functional impairment as one of the diagnostic criteria for diagnosis of many psychiatric disorders [3], but without specifying how this is to be measured. This is the first study to report on the outcomes of a well validated measure of parent report of child functioning secondary to emotional or behavior problems specifically in a normal group of children. Functional impairment in important domains of life can result in adverse outcomes (e.g., elevated risk of death and physical injury and also a significant reduction of functioning in major life activities like self-care and safe behaviors, self-efficacy, family and peer relations, and academic performance) [4].

Despite the importance of learning about the importance of understanding the impact of mental health on function in normal populations, we have not identified either a measure validated for this purpose, nor any information on descriptives of function relative to mental health concerns in this age group. This information is essential as a baseline to use as a comparator with functional impairment relative to clinical populations with various specific disorders. Such a standard would allow us to know “how normal is normal” without making the assumption that control populations are inherently free either of distress or free of functional impairment secondary to distress. Acquiring data on functional impairment secondary to emotional distress would also facilitate epidemiological studies comparing children from different socio-economic backgrounds, cultures and age groups.

The Weiss Functional Impairment Rating Scale (WFIRS) [5] is a useful measure in clinical contexts and treatment outcome studies. The WFIRS was designed to measure functional impairment in Attention Deficit Hyperactivity Disorder (ADHD) specifically, but recent validation studies looking at other clinical populations have demonstrated that it is not ADHD specific [6]. The WFIRS has been translated into 18 languages and has both parent- and self-report forms. This measure evaluates seven functional domains by including 69 items for the self-report form and six domains with 50 items for the parent-report form. To our knowledge, there are three published studies [6–8] that assessed the psychometric properties of self-report form of the WFIRS (WFIRS-S). The WFIRS-P has been psychometrically investigated using varying populations in Canada [9], China [10], Turkey [11], Germany [12], Thailand [13] and in a large sample drawn from multiple research studies conducted in Europe, North America and Australia [14]. In Weiss et al. [9], the WFIRS-P demonstrated robust internal consistency and good convergent validity. The validation study on the Turkish version of the WFIRS-P indicated that the WFIRS-P has strong internal consistency and test-retest reliability, and good convergent and discriminant validity in a group of children with ADHD and healthy children [11]. The study on the German adaptation of the WFIRS-P in a clinical sample of children with externalizing behaviors (ADHD or Oppositional Defiant Disorder (ODD)) found factorial validity, reliability, and divergent validity for the WFIRS-P [12]. In Gajria et al. [14], the validity and reliability of the WFIRS-P were assessed in a sample of children and adolescents with ADHD and the WFIRS-P demonstrated robust internal consistency and test-retest reliability, convergent validity with measures of ADHD and functioning, and responsiveness to change.

The WFIRS was designed to measure the impact of emotional and behavioral problems on functional impairment. This is different to functional impairment as an absolute trait in its own right. Functional skills as a fixed trait for that child (which may be impacted by intellectual and other factors) is different to functional impairment driven by psychopathology, especially for ADHD which can be well controlled. As a result, the WFIRS is sensitive to treatment effects and change over time [15]. However, we did not find any research measuring how emotional and behavioral factors in normal children impact their functioning. All children face some degree or another of emotional or behavioral challenges, therefore the use of the WFIRS in this sample allows us to determine the baseline for this effect in a normative population to use for comparison with clinical samples.

The objective of this study is to assess the descriptive and psychometric properties of a Persian translation of the WFIRS-P in a non-clinical school age sample of children in grades 1–6. More precisely, the current study aims to assess the descriptive characteristics, internal consistency, test-retest reliability, and interdomain correlations of the WFIRS-P.

## Method

### Aims

The aim of this study was to evaluate the descriptive characteristics, internal consistency, test-retest reliability, and interdomain correlations of the WFIRS-P in a normal sample of Iranian children.

### Development process of the Persian form of the WFIRS-P

To evaluate the validity and reliability of the Persian version of the WFIRS-P, written permission was obtained from the developer of the scale. The scale was translated to Persian by a Ph.D. student in clinical psychology who had a good and adequate comprehension of the English language and research experience with ADHD. The back translation of the measure was done by a clinical psychologist and researcher in ADHD who was fluent in both languages. The fidelity of the items was confirmed by the developer of the scale. To confirm the face validity of the items, the final Persian version was reviewed by a number of university students.

### Measures

#### Weiss functional impairment rating scale- parent-report form (WFIRS-P)

The WFIRS-P is a parent report scale which assesses functional impairment across varying domains of life. The WFIRS-P uses a 0 (never or not at all) to 3 (very often or very much) response scale. Additionally, items can be rated as “not applicable”. The scale consists of 6 domains: Family (10 items. e.g., “Having problems with brothers & sisters”), School (10 items. e.g., “Makes it difficult to keep up with schoolwork”), Life skills (10 items. e.g., “Excessive use of TV, computer, or video games”), Self-concept (3 items. e.g., “My child feels bad about himself/herself”), Social activities (7 items. e.g., “ Being teased or bullied by other children”), Risky activities (10 items. e.g., “Easily led by other children (peer pressure)”). A total score and a score for each domain can be calculated using the mean of all items excluding those rated not applicable, ranging from 0 (never) to 3 (very much). This scoring method makes it possible to compare the domains and provides a score that is specific to the functional impairment related to that individual. In clinical assessment, any domain is considered impaired if it includes two items with rating 2 (much), or one item with rating 3 (very much).

### Participants

Participants of the current study were parents of 282 male and female students. The students (see Table 1) recruited from public elementary schools (grades 1 to 6) in Shiraz, Iran.

**Table 1:** Participant Characteristics (*n* = 282)

Characteristic	
Sex (%)	
Male	138 (48.9%)
Female	144 (51.1%)
Grade (%)	
First	14 (5%)
Second	36 (12.8%)
Third	62 (22%)
Fourth	49 (17.4%)
Fifth	61 (21.6%)
Sixth	60 (21.3%)
Grade by age appropriate	
First	Age 6–7
Second	Age 7–8
Third	Age 8–9
Fourth	Age 9–10
Fifth	Age 10–11
Sixth	Age 11–12

### Procedure

The parents of the children approved and signed the informed consent form. The parents were sent a letter containing essential information about the purpose of the study and instructions on how to fill out the WFIRS-P. The questionnaire was completed at home by parents. In order to evaluate the test-retest reliability of the WFIRS-P, 28 parents repeated completion of the scale two weeks after the first administration and the scale was completed at home by the same informant. Each student in the sample of test-retest received two identical stickers with a random number on them. We asked them to put one sticker on the test form and keep the other one in his/her backpack for the retest form. We randomly distribute the stickers between the participants and nobody knows who is who. Children were instructed to bring back all completed materials. The data collection was anonymous and the scales were collected by independent psychology students.

### Statistical analysis

IBM SPSS Statistics 22 program was used for all analyses. All statistical analyses were conducted with a significance level of .05. Descriptive statistics including mean and standard deviation were used to examine WFIRS-P items and domains. Cronbach’s alpha coefficients were used to assess the internal consistency of the WFIRS-P for each domain and the total scale. Cronbach’s alpha coefficient higher than 0.7, 0.8, and 0.9, respectively, is considered acceptable, good, and excellent [16]. Pearson correlation coefficients were calculated to measure the test-retest reliability of the WFIRS-P and the interdomain correlations on the scale. The acceptable level for the test-retest reliability coefficient is usually considered greater than or equal to 0.70 [17]. Correlation coefficients were evaluated in this way: negligible correlation (0.00 to ±0.30), low (±0.30 to ±0.50), moderate (±0.50 to ±0.70), high (±0.70 to ±0.90), and very high (±0.90 to ±1.00) [18].

## Results

### WFIRS-P Descriptives

Table 2 depicts the means and SDs of each item and domain in the WFIRS-P. The mean total score of the WFIRS-P was 0.27 (*SD* = 0.20). Additionally, life skills had the highest rated impairment (*M* = 0.50, *SD* = 0.37) and risky activities had the lowest (*M* = 0.07, *SD* = 0.11). The mean score obtained for the various domains allow for domain to domain comparison. Going from highest mean scores to lowest mean scores, we found the domains were rank ordered as follows: life skills, family, self-concept, school, social activities, and risky activities. Excessive use of screens, keeping clean, brushing teeth, brushing hair, bathing, etc., and problems with eating (picky eater, junk food) from the life skills domain had the highest rated impairments (*M* = 0.66–1.16) from the whole scale. Being involved with the police, smoking cigarettes, taking illegal drugs, and sexually inappropriate behaviour from the risky activities domain had the lowest (0). This reflects that looking at the population as a whole, these items are infrequent and so have low scores at the population level, which is not to say that when present they are not seriously impairing.

**Table 2:** Item Analysis of WFIRS-P items

		Mean (SD)	0 (Never or not at all)	1 (sometimes or somewhat)	2 (often or much)	3 (very often or very much)	NA (not applicable)
A	Family	.28 (.32)					
1	Having problems with brothers & sisters	.48 (.70)	61.7	33.7	2.5	1.4	0.7
2	Causing problems between parents	.22 (.48)	79.8	18.8	0.7	0.7	0
3	Takes time away from family members’ work or activities	.60 (.71)	51.4	39.7	7.4	1.1	0.4
4	Causing fighting in the family	.27 (.53)	76.2	21.3	1.8	0.7	0
5	Isolating the family from friends and social activities	.10 (.34)	90.8	8.2	1.1	0	0
6	Makes it hard for the family to have fun together	.11 (.40)	91.5	7.1	1.1	0	0.4
7	Makes parenting difficult	.22 (.56)	83.3	13.5	1.4	1.8	0
8	Makes it hard to give fair attention to all family members	.20 (.47)	82.6	15.2	1.8	0.4	0
9	Provokes others to hit or scream at him/her	.29 (.55)	75.5	20.9	2.8	0.7	0
10	Costs the family more money	.29 (.52)	74.5	22.3	3.2	0	0
B	School	.22 (.27)					
	Learning	.49 (.57)					
1	Makes it difficult to keep up with schoolwork	.58 (.79)	56.7	33.3	6.0	3.5	0.4
2	Needs extra help at school	.49 (.71)	62.1	29.4	6.4	2.1	0
3	Needs tutoring	.44 (.65)	63.1	31.2	4.3	1.4	0
4	Receives grades that are not as good as his/her ability	.44 (.75)	67.7	24.5	4.6	2.8	0.4
	Behavior	.05 (.14)					
1	Causes problems for the teacher in the classroom	.11 (.40)	91.1	7.4	0.7	0.7	0
2	Receives “time-out” or removal from the classroom	.05 (.24)	95.4	4.3	0.4	0	0
3	Having problems in the school yard	.06 (.26)	94.3	5.3	0.4	0	0
4	Receives detentions (during or after school)	.03 (.16)	97.5	2.5	0	0	0
5	Suspended or expelled from school	.01 (.10)	98.9	1.1	0	0	0
6	Misses classes or is late for school	.01 (.12)	98.6	1.4	0	0	0
C	Life skills	.50 (.37)					
1	Excessive use of TV, computer, or video games	.84 (.83)	39.4	42.2	13.8	4.6	0
2	Keeping clean, brushing teeth, brushing hair, bathing, etc.	1.16 (.97)	8.9	25.9	35.1	29.4	0.7
3	Problems getting ready for school	.30 (.58)	74.8	20.9	3.2	1.1	0
4	Problems getting ready for bed	.45 (.67)	63.8	29.4	5.0	1.8	0
5	Problems with eating (picky eater, junk food)	.66 (.83)	52.8	33.0	9.6	4.6	0
6	Problems with sleeping	.37 (.66)	71.3	22.3	4.6	1.8	0
7	Gets hurt or injured	.40 (.58)	63.8	33.0	2.5	0.7	0
8	Avoids exercise	.16 (.46)	86.5	11.3	1.4	0.7	0
9	Needs more medical care	.20 (.47)	82.3	15.6	1.8	0.4	0
10	Has trouble taking medication, getting needles or visiting the doctor/dentist	.46 (.71)	64.5	28.0	5.3	1.8	0.4
D	Self-concept	.25 (.47)					
1	My child feels bad about himself/herself	.19 (.55)	86.2	10.6	1.8	1.1	0.4
2	My child does not have enough fun	.28 (.60)	77.7	18.1	3.2	0.7	0.4
3	My child is not happy with his/her life	.29 (.64)	78.7	16.7	2.5	1.8	0.4
E	Social activities	.18 (.28)					
1	Being teased or bullied by other children	.24 (.55)	80.1	16.3	2.5	1.1	0
2	Teases or bullies other children	.13 (.37)	87.9	11.0	1.1	0	0
3	Problems getting along with other children	.18 (.45)	84.0	14.5	0.7	0.7	0
4	Problems participating in after-school activities (sports, music, clubs)	.21 (.53)	84.0	12.4	2.5	1.1	0
5	Problems making new friends	.21 (.48)	81.9	16.3	1.1	0.7	0
6	Problems keeping friends	.18 (.47)	85.1	12.8	1.4	0.7	0
7	Difficulty with parties (not invited, avoids them, misbehaves)	.09 (.31)	92.2	7.1	0.7	0	0
F	Risky activities	.07 (.11)					
1	Easily led by other children (peer pressure)	.33 (.57)	71.3	25.2	2.8	0.7	0
2	Breaking or damaging things	.13 (.42)	89.7	8.5	1.1	0.7	0
3	Doing things that are illegal	.05 (.24)	96.1	3.2	0.7	0	0
4	Being involved with the police	.00 (.00)	100	0	0	0	0
5	Smoking cigarettes	.00 (.00)	100	0	0	0	0
6	Taking illegal drugs	.00 (.00)	100	0	0	0	0
7	Doing dangerous things	.03 (.20)	97.2	2.5	0.4	0	0
8	Causes injury to others	.01 (.12)	98.6	1.4	0	0	0
9	Says mean or inappropriate things	.10 (.43)	92.9	5.0	1.4	0.4	0.4
10	Sexually inappropriate behaviour	.00 (.00)	100	0	0	0	0

*WFIRS-P* = Weiss Functional Impairment Rating Scale-Parent Report, *SD* = standard deviation

### Internal consistency

The internal consistency of the WFIRS-P were acceptable (α’s = 0.72–0.74) for the school, self-concept, and social activities domains and were good for the family domain (α = 0.81) and total scale (α = 0.88) (see Table 3).

**Table 3:** Internal Consistency for WFIRS-P (*n* = 282)

WFIRS-P	Number of items	α
Family	10	0.81
School	10	0.74
Life skills	10	0.62
Child’s Self-concept	3	0.72
Social activities	7	0.72
Risky activities	10	0.42
Total	50	0.88

*WFIRS-P* = Weiss Functional Impairment Rating

Scale-Parent Report

α = Cronbach’s alpha coefficient

### Test-retest reliability

The results of the test-retest reliability of the WFIRS-P domains and total scale are reported in Table 4. The WFIRS-P was completed two weeks after the first administration by the same informant. The test-retest coefficient exceeded the acceptable value for the school and risky activities domains and total scale (*r* = 0.74–0.90). For the family, life skills, self-concept, and social activities domains, the correlation coefficients were less than the standard magnitude (*r* = 0.60–0.68).

**Table 4:** Means and SDs of the WFIRS-S domains and total scale in test and retest and test–retest reliability coefficients (*n* = 28)

WFIRS-S	Test		Retest		
	Mean	SD	Mean	SD	*r*
Family	.24	.36	.33	.49	0.66^*^
School	.20	.24	.25	.23	0.74^*^
Life skills	.47	.31	.58	.32	0.68^*^
Self-concept	.24	.38	.26	.53	0.61^*^
Social activities	.19	.26	.19	.32	0.60^*^
Risky activities	.06	.12	.10	.21	0.90^*^
Total	.24	.19	.30	.26	0.77^*^

*WFIRS-P* = Weiss Functional Impairment Rating Scale-Parent Report

*SD* = standard deviation, *r* = Pearson’s correlation coefficient, *p < .0

### WFIRS-P domain and total score correlations

Displayed in Table 5, all the correlations between the WFIRS-P domains with each other were statistically significant (*p* < 0.01) and ranged from negligible (*r* = 0.19–0.29) to moderate (*r* = 0.58–0.59). Additionally, each of the WFIRS-P domains showed moderate to high and statistically significant correlation with the total scale (*r* = 0.52–0.81, *p* < 0.01).

**Table 5:** Relationship between WFIRS-P domains with each other and with the summary scale (*n* = 282)

WFIRS-P	Family	School	Life skills	Self-concept	Social activities	Risky activities	Total
Family	_						
School	.40^*^	_					
Life skills	.59^*^	.38^*^	_				
Self-concept	.29^*^	.31^*^	.38^*^	_			
Social activities	.45^*^	.40^*^	.43^*^	.38^*^	_		
Risky activities	.58^*^	.40^*^	.46^*^	.19^*^	.35^*^	_	
Total	.81^*^	.69^*^	.78^*^	.52^*^	.68^*^	.65^*^	_

*WFIRS-P* = Weiss Functional Impairment Rating Scale-Parent Report

Pearson’s correlation analysis, *p < .01

### The effect of age on the WFIRS-P total scale

There was no significant difference in the WFIRS-P total scale among ages (*F* (5,276) = 1.375, *p* = 0.234, partial*η*^*2*^ = 0.024).

## Discussion

In this study, the descriptive and psychometric properties of the Persian version of the WFIRS-P were assessed in a sample of normal Iranian children in grades 1–6. This is the first study in Iran assessing the descriptive and psychometric properties of a Persian scale of functional impairment in children. Based on the descriptive findings of this study, the mean total score of the WFIRS-P was 0.27 which means that the majority of parents see their children as having mimimal difficulty. This finding was not unexpected in a normal sample of children and is consistent with previous studies on the WFIRS-P [11, 19] and one study on the WFIRS-S [7]. In Thompson et al.’s study [19] in an ADHD group and a non-ADHD control group, the mean total score of the WFIRS-P was 0.37 in the control group and in Tarakçıoğlu et al.’s study [11] in a sample of children with ADHD and healthy children, the mean total score of the scale was 0.15 in healthy group. In Hadianfard et al.’s study [7] on the WFIRS-S in a non-clinical sample of adolescents the mean total score was 0.31 for the scale. In Gajria et al.’s study [14] on the WFIRS-P, the mean total score in the first and second random split half-sample of children and adolescents with ADHD was 1.03 and 1.01, respectively. Among the WFIRS-P domains and items, the life skills domain overall and 3 items in particular from this domain had the highest mean scores. This suggests that life skills are a common developmental challenge for parents and they experience the most concern about failure to meet expectations in this area, especially “excessive use of TV, computer, or video games”, “keeping clean, brushing teeth, brushing hair, bathing, etc.”, and “problems with eating (picky eater, junk food)” however the higher scores in the life skills do not necessarily have a higher level of clinical impact on the well-being of the child. This is consistent with Thompson et al.’s study [19] which showed the life skills has the highest rated impairment with a mean score of 0.52 and inconsistent with Tarakçıoğlu et al.’s study [11] which found the self-concept has the highest rated impairment (0.26) and the life skills has the second rank (0.23). This is also of particular interest in that in treatment outcome studies, life skills has had a relatively sluggish response to treatment [15].

In Gajria et al.’s study [14] on a clinical sample of children and adolescents with ADHD, the school domain of the WFIRS-P had the highest mean score. This difference between the current study and Gajria et al.’s study [14] represents the difference between a normal sample and a clinical ADHD sample in functional impairment, demonstrating both that the measure is appropriate to both clinical and non-clinical population and sensitive to the specific functional impairments that are associated with those domains. Further study, for example, might look at whether an autism population has selective impairment in the social domain, or whether a depressed population had selective impairment in the self-concept domain. Additionally, the results of our study showed that the risky activities domain and 4 items from this domain including “being involved with the police”, “smoking cigarettes”, “taking illegal drugs”, and “sexually inappropriate behaviour” have the lowest mean scores in normal children. Our finding that risky activities has relatively low scores is consistent with Thompson et al.’s and Tarakçıoğlu et al.’s studies [11, 19] on the WFIRS-P in the healthy group of their sample and in line with Hadianfard et al.’s study [7] on the WFIRS-S in normal adolescents. In Gajria et al.’s study [14] on the WFIRS-P, children and adolescents with ADHD showed the lowest mean score in the risky activities domain. This is consistent with the intent of inclusion of the risky activities domain as a way of capturing less frequent, but nonetheless very salient items of high risk behavior that is functionally impairing. The absolute value of the scores should not be considered as an indication of actual impairment. For example, ratings for risky activities are low in a normal population because they are rare but their impact on the life of an individual when present can be very high. It is also of interest that in outcome studies of clinical trials using the WFIRS, Risky Activities has shown robust response to treatment despite floor effects [15].

Validation of the measure in a population of normal, Iranian children indicates that the WFIRS-P can be used to identify functional skills both in populations studies, healthy controls and across cultures. This should allow further research into the specific relative functional strengths and weaknesses associated both with ADHD and with other disorders, as well as more information as to the functional profile that is specific to ADHD at different levels of severity.

Cronbach’s alpha coefficients were acceptable to good for the domains and total scale of the WFIRS-P, with lower internal consistency found for the life skills and risky activities. The lower internal consistency for the life skills and risky activities domains is consistent with the previous studies [9, 11, 14]. In Gajria et al.’s study [14], the life skills and risky activities domains of the WFIRS-P had lower internal consistency relative to other domains. In Weiss et al.’s and Tarakçıoğlu et al.’s studies [9, 11], the risky activities domain of the WFIRS-P indicated the lowest internal consistency. The high-risk behaviors (from peer pressure to sexually inappropriate behavior) of this domain would be expected to be rare in population samples, thus lowering the internal consistency, although this would have to be also be tested in vulnerable but non-clinical samples that might be found, for example, in inner city, low income areas.

Test-retest reliability coefficients were more than the acceptable value for the total scale, school and risky activities domains. This demonstrates the capacity for reliable parent report of the impact of emotional and behavioral issues on function in children. Test-retest correlations approximated the acceptable value for the family and life skills domains and were lower for the self-concept and social activities domains. Self-concept domain of the WFIRS-P includes items that require parents to describe the subjective feelings of the child about himself/herself. Additionally, the social activities domain of the WFIRS-P consists of items many of which are related to the child’s social relationships with his/her friends and peers. As a result, assessing the child in the self-concept and social activities domains require parents to recognize their child’s feelings and his/her relationships with other children. Parents may be less well informed in these areas relative to their recognition in more objective functional domains.

In this study, the small to moderate correlation of the domains with each other suggests that the scale successfully captures distinct domains of impairment while the high correlation of the domains with the scale as a whole suggests it retains its integrity as a measure of functional impairment overall. The family and self-concept domains showed the highest and lowest correlation with the total scale, respectively. Generally, these results are consistent with the prior studies on the WFIRS-P [9, 11].

## Conclusion

This is the first validation study on the Persian version of the WFIRS-P. This study demonstrated that the WFIRS-P has adequate psychometric properties in Iranian non-ADHD children. The validation of the measure among normal children should allow for use of the WFIRS-P to obtain comparative information among children in other cultures and settings. For example, report by parents on risky activities of Iranian children shows this to be quite rare, which might be very different to children or adolescents in inner city environments or in children living in disadvantaged circumstances. This is the first validation of a measure of functional impairment appropriate to a healthy population of children in the Middle East. Understanding the functional impairment of clinical samples is only possible if we know the level of functional impairment or skill in the population at large.

The WFIRS was developed to measure the impact of emotional and behavioral problems on how children function. All children, not just those with a psychological disorder, have some degree of emotional and behavioral problems that can impair them across differing domains of life. Therefore, evaluating functional impairment in normal children is useful to address any impairments to help the child function more optimally. Altogether, the Persian version of the WFIRS-P has been found to be an adequate measure of functional impairment in typically developing Iranian children.

There are several important clinical implications to these findings. The validation of the WFIRS in both normative and clinical samples further contributes to our understanding of the robust psychometrics of the measure. In addition, similar internal consistency, test retest and (insert other tests used) indicates that these characteristics of function are consistent to both clinical and non-clinical populations. Lastly, in future outcome studies of the WFIRS on clinical outcomes for intervention the results can be compared to WFIRS scores obtained in a normative population.

This study demonstrates that the WFIRS maintains the same robust psychometric properties in non-clinical as well as clinical populations. We have found modest differences between a normal Persian population and similar studies done in other countries, which may reflect cross cultural differences. This will be an interesting area for future research. The WFIRS appears to be an effective measure to use in obtaining more information about function in different cultures, age groups, informants, and genders. Large scale normative studies are needed to provide us with a better understanding of functional strengths and weaknesses of youth.

## Abbreviations

ADHD: Attention Deficit Hyperactivity Disorder
DSM-5: Diagnostic and Statistical Manual of Mental Disorders, Fifth Edition
ODD: Oppositional Defiant Disorder
WFIRS: Weiss Functional Impairment Rating Scale
WFIRS-P: Weiss Functional Impairment Rating Scale-Parent Report Form
WFIRS-S: Weiss Functional Impairment Rating Scale-Self-Report Form
